# A new method for optimal placement of tumor treating fields electrodes

**DOI:** 10.1093/noajnl/vdag148

**Published:** 2026-06-03

**Authors:** Konstantin Weise, Nikola Mikic, Fang Cao, Eric T Wong, Thomas R Knösche, Axel Thielscher, Anders Rosendal Korshøj

**Affiliations:** Department of Clinical Medicine, Aarhus University, Aarhus N, Denmark; Methods and Development Group “Brain Networks”, Max Planck Institute for Human Cognitive and Brain Sciences, Leipzig, Germany; Institute of Measurement, Control and Automation Engineering, Leipzig University of Applied Sciences (HTWK), Leipzig, Germany; Department of Clinical Medicine, Aarhus University, Aarhus N, Denmark; Department of Neurosurgery, Aarhus University Hospital, Aarhus N, Denmark; Danish Research Centre for Magnetic Resonance, Department of Radiology and Nuclear Medicine, Copenhagen University Hospital Amager and Hvidovre, Hvidovre, Denmark; Danish Research Centre for Magnetic Resonance, Department of Radiology and Nuclear Medicine, Copenhagen University Hospital Amager and Hvidovre, Hvidovre, Denmark; Brown University Health Cancer Institute & The Warren Alpert Medical School of Brown University, Providence, Rhode Island, USA; Methods and Development Group “Brain Networks”, Max Planck Institute for Human Cognitive and Brain Sciences, Leipzig, Germany; Section for Magnetic Resonance, Department of Health Technology, Technical University of Denmark, Lyngby, Denmark; Department of Clinical Medicine, Aarhus University, Aarhus N, Denmark; Department of Neurosurgery, Aarhus University Hospital, Aarhus N, Denmark

**Keywords:** electrode optimization, glioblastoma, NovoTAL, SimNIBS, tumor treating fields

## Abstract

**Background:**

Tumor Treating Fields (TTFields) provide a noninvasive treatment option for newly diagnosed glioblastoma. While electrode placement is considered important for treatment efficacy, current clinical planning relies on a proprietary and undisclosed software (NovoTAL). This study investigates a new computational approach for optimizing TTFields electrode placement and compares it with the current clinical standard.

**Methods:**

We developed a computational pipeline integrating patient-specific anatomical data to optimize electrode configurations in five representative glioblastoma cases spanning diverse tumor locations and sizes. Two optimization strategies were investigated: one maximizing electric field intensity at the tumor and another increasing coverage of the surrounding brain while maintaining tumor intensity. Results were compared with electrode placements generated by NovoTAL. Additional simulations with artificial tumors assessed the effects of tumor size and location.

**Results:**

Optimized electrode placements increased tumor electric field intensity by 18%-34% compared with the clinical standard. Coverage-weighted optimizations achieved broader field coverage with minimal reduction in tumor intensity. Smaller and surface-adjacent tumors benefited most from optimization. Extensive randomized placement analyses demonstrated the superior performance of the optimized configurations. Artificial tumor models showed consistent improvements across a range of tumor locations and sizes.

**Conclusions:**

Personalized optimization of TTFields electrode placement improved simulated electric field metrics within tumors and adjacent brain regions compared with the current clinical planning approach. These findings support the potential of patient-specific computational planning and the future development of adaptive, automated TTFields planning strategies. The clinical significance of the observed field improvements remains to be established in prospective studies.

Key Points

- Optimized TTFields array placement enhanced field intensity by 18%-34% vs. clinical standard.
- Optimized TTFields planning improved field coverage in tumor-adjacent regions.
- Optimized TTFields planning outperformed standard methods and random placement.

Importance of the Study

TTFields are used as adjuvant therapy for glioblastoma. However, current individualized treatment planning relies on proprietary, undisclosed, and clinically unvalidated software, limiting transparency, optimization, and innovation in the field. This study introduces an individualized, semi-automated, and open-source method for optimal electrode placement based on standard MRI data, addressing a critical need for validated and adaptable planning tools. Our approach increased field intensity in tumors by 18%-34% and achieved broader coverage compared to the standard clinical tool (NovoTAL), without compromising therapeutic strength. The method also consistently outperformed extensive random electrode placements across diverse tumor types and sizes, highlighting its robustness and ability to achieve true optimal configurations. Open-source availability enhances reproducibility and clinical translation, representing a step toward more transparent and personalized TTFields planning. While the present findings are based on simulations, they provide a foundation for future studies investigating whether improved field metrics translate into clinical benefit for patients.

The management of glioblastoma continues to pose significant challenges, motivating the development of new therapeutic strategies. One such approach is Tumor Treating Fields (TTFields), a noninvasive treatment that employs alternating electric fields (E-fields) to disrupt cancer cell proliferation. First introduced by Kirson et al in early clinical trials,^1^ TTFields have demonstrated efficacy against solid cancers,^2^ including newly diagnosed glioblastoma,^3^ non-small cell lung cancer,^4^ and pancreatic adenocarcinoma.^5^ The electric fields are thought to exert forces on charged or polarizable molecules within dividing cells, disrupting critical structures, including the mitotic spindle and membrane proteins, ultimately causing cancer cell death.^6^^,^^7^

The intensity of TTFields is believed to constitute an important dosimetric relationship with treatment efficacy.^8^ Specifically, preclinical studies have demonstrated enhanced anti-neoplastic effects at increased field intensities across diverse cancer types.^1^^,^^9^ Moreover, post hoc analyses of data from the EF-14 trial^3^ demonstrate an association between high field intensities in the tumor, longer overall survival,^10^ and a reduced risk of local recurrence.^11^ This dose-response correlation has prompted the development of the proprietary software NovoTAL (Novocure, Ltd.), which is used together with the TTFields technology Optune (Novocure, Ltd.), to prepare an optimized electrode layout for each patient. This concept compares to the principle of dose-planning in radiation oncology and is based on the fact that the placement of TTFields electrodes on the patient’s scalp directly influences the distribution and intensity of the electric fields, and hence expectedly impacts the therapeutic outcome. To achieve maximum clinical benefit of TTFields across cancer types, methods are needed to ensure optimal electrical energy deposition in the regions of interest.^12^

The main objective of electrode planning, therefore, lies in determining individual electrode configurations that ensure optimum field intensity in the tumor. For glioblastoma, which infiltrates the adjacent brain tissue diffusely, maintaining high field intensities at tumor sites and adjacent regions bearing microscopic disease is also important.

Currently, however, NovoTAL is the only clinical tool available for TTFields electrode array planning. Its workflow is based on the morphometric inputs from structural MR images of the patient’s head (Supplementary Figure S1). The specific details of the underlying algorithm have not been publicly described. In addition, the output is limited to a set of predefined standardized layouts. While this approach has enabled clinical implementation of TTFields planning, ­comparisons with alternative optimization strategies and systematic evaluations of its impact on electric field optimization remain limited. These aspects motivate the exploration of complementary planning approaches.

Recent advances in electric field modeling of TTFields have established a detailed, multiscale understanding of how alternating electric fields interact with tissue and cellular structures. As summarized by Wenger and colleagues,^7^^,^^13^ state-of-the-art approaches employ volume conductor models solved using the finite element method under the electro-quasistatic approximation, enabling simulation of electric field distributions within patient-specific head models. This allows individualized electric-field estimates that reflect anatomical and biophysical variability across patients. Korshøj and colleagues^14^^,^^15^ extended these methods to analyze how tumor position, conductivity distribution, and skull geometry influence field strength and how targeted craniectomy or optimized electrode placement can enhance local TTFields intensity. Furthermore, related methodological developments by Thielscher and colleagues in realistic head modeling for noninvasive brain stimulation^16^ have directly informed TTFields dosimetry pipelines.

In a recent study, we proposed a generalized optimization approach based on computational modeling and simulation of the electric field distributions to optimize electrode placement across diverse technologies, including Transcranial Electrical Stimulation (TES), Temporal Interference Stimulation (TIS), Electroconvulsive Therapy (ECT), and TTFields.^17^ These simulations utilize advanced algorithms and finite element analysis to optimize the electric fields within complex anatomical structures to find optimal electrode arrangements by allowing free movement of the electrode arrays. By modeling patient-specific anatomical features based on standardized MRI data, it is possible to tailor electrode arrangements to ensure optimal therapeutic efficacy, such as maximal TTFields intensity within the tumor volume to enhance cytotoxic effects.^18^

In this study, we applied the electrode optimization methodology of Weise et al^17^ to TTFields, illustrated in Figure 1A, and evaluated its potential compared to the current clinical standard. For this purpose, we selected 5 representative patients with different tumor locations and sizes, and we compared the optimization method of Weise et al^17^ to the electrode positions using the NovoTAL planning software, which corresponds to the current clinical standard. We then juxtaposed the resulting electric fields in the tumor region from either method. Furthermore, we tested the method against extensive randomized array positioning to document robustness and true optimization across diverse tumor types and locations.

**Figure 1.**
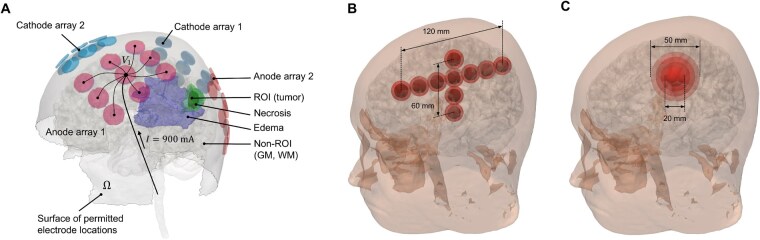
(A) Visualization of the TTFields electrode arrays on the patient’s head, together with the region-of-interest (ROI) containing the tumor and the “Non-ROI” containing the rest of the brain. The skin surface is restricted to locations where the electrode arrays can be placed, ie not over the eyes and ears of the patients; (B) modified *ernie* head model with different artificial tumor locations in superior-inferior (S-I) and anterior-posterior (A-P) direction and (C) tumor size to systematically study the impact of electrode optimization compared to current standard treatment strategies using TTFields.

## Methods

The optimization pipeline used to determine the optimal electrode placements for TTFields is implemented in SimNIBS v4.5.^19^ All head models used in this study were created with T1 and T2 images using the CHARM pipeline.^20^

We studied real application scenarios on 5 patients representing a realistic variety of tumor morphologies and locations. Tissue volumes of edema, necrosis, and residual tumor were manually segmented using MRI patient data based on a standardized brain tumor protocol for MRI.^21^ Following the Declaration of Helsinki, the study was reviewed and approved by the Central Denmark Region Committee for Health Research Ethics (68928/1-10-72-214-19) and the Danish Data Protection Authority. Written consent was obtained from the trial participants for using non-identifiable imaging for future silico studies.

The electric conductivities for the pathological tissues were $\sigma_{\mathrm{res}.\mathrm{tumor}}=0.24\;S/m$, $\sigma_{\mathrm{necrosis}}=1.0\;S/m$, and $\sigma_{\mathrm{edema}}=1.0\;S/m$.^14^^,^^15^^,^^22^ The electrical conductivities of the remaining tissues are given in Supplementary Table S1.

To systematically investigate the influence of tumor size and location, artificial tumors were added to the *ernie* head model, which is part of the example dataset of SimNIBS (https://simnibs.github.io/simnibs/build/html/dataset.html). The artificial tumor had a diameter of 20 mm and contained a necrotic inner part with a diameter of 14 mm. Its location was varied in longitudinal (superior to inferior, S-I) and sagittal (anterior to posterior, A-P) direction as shown in Figure 1B. Additionally, the diameter of the tumor was expanded from 20 mm to 50 mm (Figure 1C).

For each patient and the *ernie* head model, the required head and tumor measures were extracted from the MRI datasets and transferred to the planning software of NovoTAL to determine the electrode positions of the current clinical standard. The software provides the suggested positions and orientations of the electrode arrays in the form of a graphical output using a general head template. The electrode configurations were then manually applied to the computational head models to calculate the associated electric field distributions using SimNIBS v4.5. In addition, the electrode positions were determined using the optimization algorithm of SimNIBS v4.5 for comparison.^17^ The algorithm determines the electrode array positions on the head that maximize a user-defined goal function. Two different goal function definitions were tested. In the first optimization approach (termed *intensity-optimized* in the following), the goal was to determine an electrode configuration that maximizes the average electric field strength in the tumor region. The second optimization approach (termed *coverage-optimized*) strived to maximize the spread of the electric field in the brain while also ensuring a field strength of 100 V/m in the tumor region. The threshold of 100 V/m was motivated by preclinical studies demonstrating anti‑mitotic effects above this intensity, widely used computational benchmarks adopting ≥100 V/m as a therapeutic threshold, and clinical‑dosimetric analyses correlating intensities above 100-110 V/cm with improved survival in GBM.^1^^,^^9^ The second approach is motivated to sustain high field intensities throughout the rest of the brain to also target diffusely infiltrating cancer cells. In this context, the goal function is defined to maximize sensitivity while minimizing specificity. By defining the tumor and the rest of the brain as separate ROIs, the sensitivity and specificity can be optimized, given the chosen threshold of 100 V/m, by allowing the electrode arrays to move freely over the head surface. Details about the implementation of the optimization algorithm are given in Weise et al.^17^ Lastly, the final electric field distributions of the different optimizations were compared to the clinical standard using the NovoTAL planning software.

## Results

**Figure 2.**
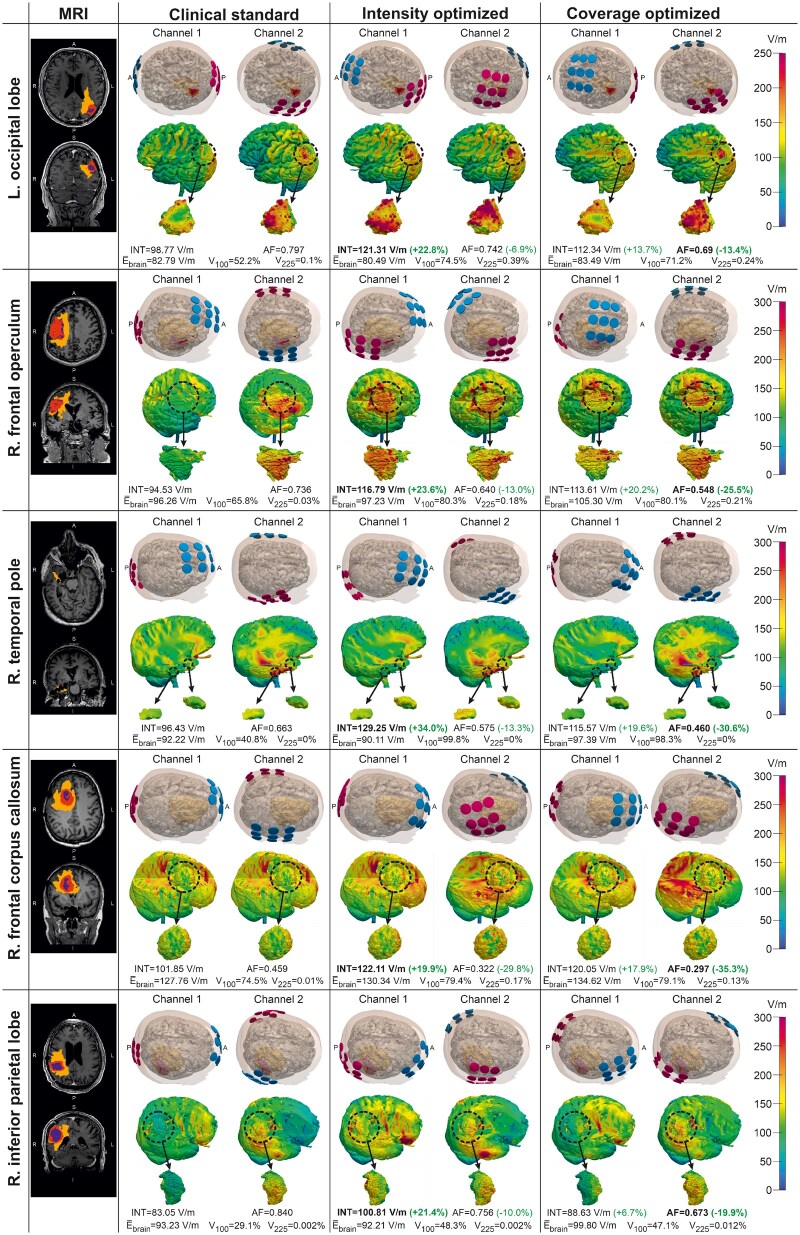
Electric field distributions created from both stimulation channels. The rows show different patients. The first column shows axial and sagittal cross sections of the tumor segmentations (blue: necrosis; red: tumor; orange: edema). The second column shows the electric field distributions created using the clinical standard from the NovoTAL planning software and the last two columns show the E-field intensity and E-field spread optimized results. Metrics: INT: average E-field magnitude in the tumor (higher is better); AF: coverage or “anti-focality” score (lower is better); ${\bar{E}}_{\mathrm{brain}}$: average E-field magnitude in the whole brain; V_100_: Relative tumor volume over 100 V/m with respect to total tumor volume; V_225_: Relative tumor volume over 225 V/m with respect to total tumor volume.

Figure 2 presents a comparison of the current clinical standard with the optimized TTFields configurations for the five patients. The corresponding electric field and coverage scores shown in Figure 2 are summarized in Table 1. Across all cases, the individual optimization consistently yielded higher average electric field strengths within the tumor region, ranging from 18% to 34%, relative to the current clinical standard. This improvement was observed for both intensity-optimized and coverage-optimized configurations. Depending on the chosen optimization criterion, either the intensity or the field coverage was enhanced, albeit with a slight tradeoff in the other metric. For example, in the patient with a tumor in the left occipital lobe (first row in Figure 2), switching from an intensity-optimized to a coverage-optimized electrode setup resulted in a tradeoff: the intensity increase attenuated from +22.8% to +13.8% relative to the clinical standard, while the coverage improved from -6.9% to -13.4%. The average tradeoff over all patients was 8.7% in intensity and 10.3% in coverage score.

**Table 1.** Comparison of the average electric field in the tumor (higher is better), the coverage score (anti-focality score, AF score, lower is better), and the relative volume of the tumor with respect to the total tumor volume in which the average electric field of the two stimulation channels exceeded values of 100 V/m between the current clinical standard and the optimized montages; values in brackets are calculated over the whole brain

	Clinical standard			Intensity optimized					Coverage optimized				
Patient	Avg. E-field in tumor (V/m)	AF score	Rel. E-field volume > 100 V/m (whole brain)	Avg. E-field in tumor (V/m)		AF score		Rel. E-field volume > 100 V/m (whole brain)	Avg. E-field in tumor (V/m)		AF score		Rel. E-field volume > 100 V/m (whole brain)
L. occipital lobe $V_{\mathrm{necrosis}}=1,412\;\mathrm{mm}³$$V_{\mathrm{tumor}}=4,682\;{\mathrm{mm}}^{3}$ $V_{\mathrm{edema}}=34,312\;\mathrm{mm}³$	98.77	0.797	52.2% (39.1%)	**121.31**	**+22.8%**	0.742	-6.9%	74.5% (32.4%)	112.34	+13.7%	**0.690**	**-13.4%**	71.2% (39.7%)
R. frontal operculum $V_{\mathrm{necrosis}}=5,988\;\mathrm{mm}³$$V_{\mathrm{tumor}}=27,067\;{\mathrm{mm}}^{3}$ $V_{\mathrm{edema}}=84,487\;\mathrm{mm}³$	94.53	0.736	65.8% (57.3%)	**116.79**	**+23.6%**	0.640	-13.0%	80.3% (44.9%)	113.61	+20.2%	**0.548**	**-25.5%**	80.1% (63.2%)
R. temporal pole $V_{\mathrm{necrosis}}=0\;\mathrm{mm}³$ $V_{\mathrm{tumor}}=124\;{\mathrm{mm}}^{3}$$V_{\mathrm{edema}}=3323\;\mathrm{mm}³$	96.43	0.663	40.8% (52.3%)	**129.25**	**+34.0%**	0.575	-13.3%	99.8% (49.8%)	115.57	+19.6%	**0.460**	**-30.6%**	98.3% (57.8%)
R. frontal corpus callosum $V_{\mathrm{necrosis}}=5399\;\mathrm{mm}³$$V_{\mathrm{tumor}}=19,599\;{\mathrm{mm}}^{3}$ $V_{\mathrm{edema}}=73,201\;\mathrm{mm}³$	101.85	0.459	74.5% (90%)	**122.11**	**+19.9%**	0.322	-29.8%	79.4% (82.5%)	120.05	+17.9%	**0.297**	**-35.3%**	79.1% (91.3%)
R. inferior parietal lobe $V_{\mathrm{necrosis}}=13,553\;\mathrm{mm}³$$V_{\mathrm{tumor}}=17,192\;{\mathrm{mm}}^{3}$ $V_{\mathrm{edema}}=67,424\;\mathrm{mm}³$	83.05	0.840	29.1% (57.3%)	**100.81**	**+21.4%**	0.756	-10.0%	48.3% (43.3%)	98.79	+18.9%	**0.673**	**-19.9%**	47.1% (59.9%)
Average	94.93	0.699	52.5% (59.2%)	**118.05**	**+24.3%**	0.607	-14.6%	76.5% (50.6%)	112.07	+18.1%	**0.534**	**-24.9%**	75.2% (62.4%)

For every patient, the volume in mm³ of necrosis, tumor, and edema region is given in the first column. Bold values highlight improved metrics for intensity and converage optimized montages with respect to the current clinical standard.

Additionally, the relative volume in the tumor area (with respect to the total tumor volume) in which the average electric field of the two stimulation channels exceeded values of 100 V/m was computed. The results are shown in Figure 2 for the individual cases and are summarized in Table 1. The relative volumes were also calculated over the whole brain, including tumor and are given in brackets in Table 1. The relative volumes exceeding a threshold of 100 V/m could be considerably increased by the optimization procedure compared to the standard placement.

**Figure 3.**
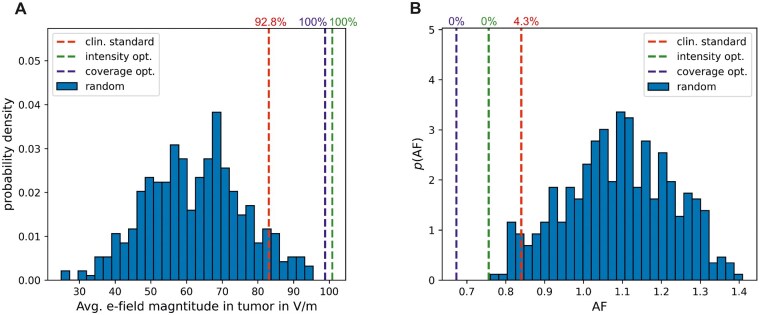
Histograms of the goal function values resulting from 200 random electrode placements for (A) E-field intensity optimization, showing the average electric field in the tumor (higher is better), and (B) optimization of the coverage score (anti-focality, AF, lower is better) from Weise et al. (2025). Results are shown for the patient with a tumor in the right inferior parietal lobe (last row in Figure 2). The histograms of the remaining patients are shown in Supplementary Figure S2 in the Supplementary Material. The scores from the clinical standard using the NovoTAL planning software are shown as red dashed lines.

In additional analysis, the performance of the optimized configurations was compared against random placements of the electrode arrays. To this end, electric fields were computed for 400 randomized array configurations for each patient. Histograms of the average electric field and coverage score for one representative patient with a tumor in the right inferior parietal lobe are shown in Figure 3. Similar histograms for the other patients are provided in the Supplementary Material (Supplementary Figure S2). These results indicate that the electrode configuration suggested by the NovoTAL planning software outperforms the average scores from random placements, while the individual optimization algorithm yields further enhancements in performance metrics.

**Figure 4.**
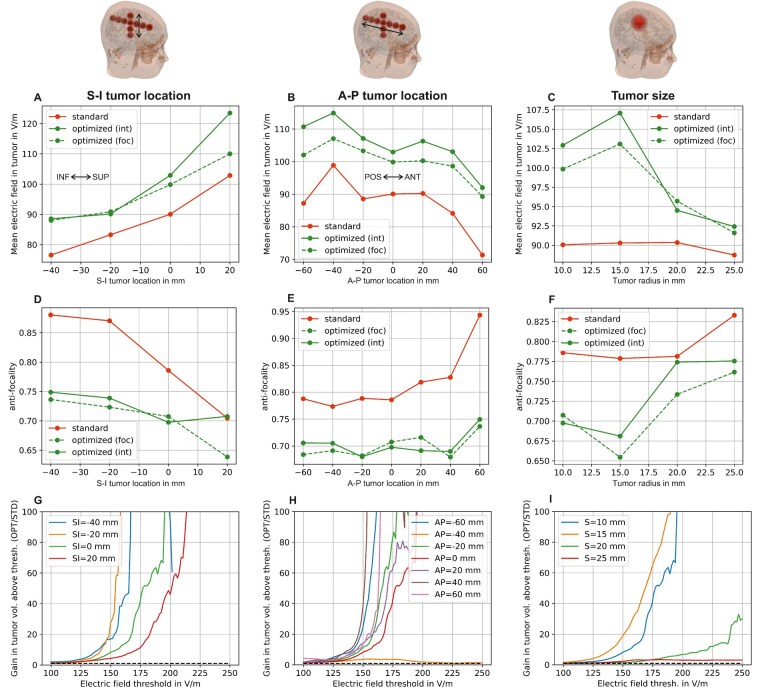
Comparison of electric field metrics between standard and optimized electrode positions for different tumor locations and sizes: (A-C) Average electric field in the tumor for S-I and A-P tumor locations, and tumor size, respectively (higher is better); (D-F) Coverage score (anti-focality, lower is better) in the brain for S-I and A-P tumor locations, and tumor size, respectively (lower is better); (G-I) Ratio of the relative volume of the tumor area with respect to the total tumor volume in which the average electric field of the two stimulation channels exceed a given electric field threshold (x-axis). The ratio is computed between intensity optimized and standard electrode positions. Values greater than 1 indicate the expected gain factor in relative tumor volume when using intensity optimization compared to the NovoTAL procedure.

The influence of tumor location and size on optimization outcomes was explored using artificial tumors modeled within the *ernie* head model. Figure 4A-C illustrates the average electric fields achieved by the NovoTAL software and the optimization algorithms for tumors as a function of tumor size as well as S-I and A-P tumor position. Notably, optimization substantially increased the absolute field strength for tumors near the surface (Figure 4A). For A-P tumor locations (Figure 4B), the optimization yielded a consistent improvement in the mean electric field strength by approximately 20% across all positions. Regarding tumor size (Figure 4C), smaller tumors (≤15 mm) exhibited a higher benefit compared to larger ones (≥20 mm) from optimized electrode placements due to enhanced targeting accuracy.

Additionally, electrode configurations optimized for electric field coverage produced comparatively higher mean electric field strengths relative to standard configurations. Figure 4D-F demonstrate that optimization focusing on field coverage resulted in significant improvements over standard electrode placements.

To further quantify these differences, partial tumor volumes exposed to electric fields exceeding specific thresholds were analyzed. The volume ratios between the optimized and standard configurations were calculated and are displayed in Figure 4G-I for different tumor locations and sizes. Ratios exceeding one indicate increased field exposure, underscoring the better performance of optimized configurations in enhancing electric field coverage within the tumor region.

## Discussion

The findings of this study demonstrate further advancement in individually optimized TTFields electrode placements with the perspective of improving therapeutic outcomes. Using computational modeling, the approach consistently achieved better electric field distributions in the tumor region compared to the current clinical standard, as implemented by the NovoTAL planning software. Specifically, the algorithm improved field coverage and enhanced the average field strength in the tumor by 18% to 34% across five representative patient cases. This improvement was further evident across various tumor locations, sizes, and configurations based on artificial head models, underscoring the robustness and adaptability of the optimization method. The results align well with recent work to improve the TTFields effects by personalizing the treatment.^8^ For instance, Korshoej et al^23^ demonstrated that patient-specific modeling improves field coverage and intensity in the glioblastoma. In that study, a manual search was used to find better electrode configurations compared to the current clinical standard, with the aim of demonstrating the possibility to improve beyond this standard. However, manual search is unfeasible in a clinical environment and also does not ensure the optimality of the solution. In contrast, numeric optimization offers a principled approach that yields optimal or close-to-optimal configurations, as we demonstrated by comparison with a large number of random configurations.

The current optimization requires approximately 4-5 hours of computation. However, as TTFields planning is typically performed between imaging and treatment initiation, this step can be executed offline without affecting the clinical workflow. Further reductions in computation time may be achievable through algorithmic refinements and parallelization.

The presented approach offers flexibility to adapt the optimization goal to the specific clinical use case—either maximizing the field in the tumor region or maintaining a balance between high field strengths in the tumor while also achieving broader field coverage. The choice of objective can be guided by the clinical context: intensity‑optimized layouts when maximizing tumor‑bed field is preferred and coverage‑optimized layouts when broader high‑field exposure of tumor‑adjacent brain is prioritized. Both strategies retain a ≥ 100 V/m floor at the tumor to maintain biologically meaningful exposure; however, prospective validation is required to map these dosimetric trade‑offs to clinical benefit. Collectively, the method enables flexible and objective optimization based on user-defined regions of interest and balanced clinical considerations.

The study highlights a clear influence of tumor size and location on electrode optimization. Smaller tumors and those located near the surface were particularly responsive to optimized placements, benefiting from more precise targeting. In the present simulation study, we observed that tumors with a radius smaller than 20 mm benefited most from the optimization. However, the targeting of the larger and more irregularly shaped tumors seen in some of the patient models was also clearly improved, highlighting the benefits of personalized optimization, including electric field simulations to address anatomical heterogeneity and complex tissue geometric profiles. This is currently not considered in standardized clinical dose-planning with NovoTAL.

Despite promising results, certain limitations of our optimization method warrant consideration. While it demonstrated robust performance in simulated scenarios and patient-specific models, its efficacy in a clinical setting remains to be validated through randomized trials. Additionally, the dual optimization criteria—field intensity in the tumor target and coverage—provide a flexible framework. However, clinical and radiological criteria for balancing these factors, eg depending on the tumor type and distribution, still need to be developed. Moreover, conductivity values for tumor and edema were fixed based on literature values. Although tissue conductivities may vary between patients and across tumor subregions, both the clinical standard configuration and the optimized layouts were evaluated under identical assumptions. Therefore, while absolute field values are affected by conductivity variability, the relative comparison between methods is expected to remain largely robust. Future studies could explore the sensitivity of the optimization to conductivity uncertainties or incorporate patient-specific estimates and larger sample sizes.

It has been shown that there is an association between clinical TTFields’ efficacy and the estimated TTFields dose,^24^^,^^25^ which, according to Ballo et al,^10^ can be defined as the product of the TTFields average power loss density in the tumor bed *and* device usage (compliance). Power loss density and electric field magnitude are equivalent and related by the electrical conductivity of the tissue. Optimization therefore also offers the possibility of reducing the current amplitude to minimize side effects, which in turn could improve compliance and increase the TTFields dose in the long run.

The patient-specific cohort in this study is limited to five cases, and, therefore, the results should be interpreted as a proof-of-concept evaluation of the proposed optimization framework. The selected patients were chosen to represent a range of tumor locations and geometries relevant for TTFields planning, allowing us to assess the behavior of the method across different anatomical scenarios. In addition, artificial tumor simulations were included to systematically explore the generalizability of the approach under controlled conditions that cannot easily be obtained from a small clinical dataset. Nevertheless, further validation in larger and more diverse patient cohorts will be necessary to fully assess the robustness and clinical applicability of the proposed planning strategy.

Moreover, our study relied on static tumor models, which do not account for dynamic changes in tumor size or shape during treatment. Adjustment of array placement based on changes in tumor volume as seen on MRI would be important. Automatic optimizations, as introduced here, could be an important component to make such adjustments feasible in clinical practice. Third, manual application of electrode configurations, as described, may introduce user-dependent variability. Therefore, automating and systematically guiding this process could streamline clinical implementation of the optimized configurations. Fourth, other metrics in addition to the field intensity, including specific absorption rate (SAR) and current density, have been shown to impact TTFields efficacy.^26^^,^^27^ SAR is a measure of energy deposition in tissue, influenced by factors such as tissue density—for example, the presence of cerebral edema around a tumor.^28^^,^^29^ Lastly, at the cellular level, the field direction relative to the direction of cancer cell mitosis has been shown to play a role.^1^^,^^7^ In preclinical research, increased TTFields efficacy was achieved using a combination of alternating orthogonal fields, yielding improved coverage of random mitotic directions. Previous studies have described alternative metrics of TTFields dose^18^ and related optimization methods^30^ to incorporate these factors in the evaluation of clinically applied electrode configurations. Future implementations of our algorithm may consider exploring these metrics in the objective function for more comprehensive optimization, although clinical validation is needed.

In addition to optimizing electrode placement, surgical approaches such as skull remodeling with cranial burr holes have been investigated to enhance TTFields intensity by mitigating the skull’s natural resistance to electrical currents.^15^^,^^31-35^ However, recent results^35^ indicate that such skull remodeling does not significantly improve treatment outcomes in recurrent glioblastoma, suggesting that dose enhancement may be less critical in this setting. Nevertheless, optimizing field distribution through surgical or planning-based methods could still hold relevance for patients with newly diagnosed glioblastoma, where TTFields efficacy may depend more on achieving higher local field intensities.

In summary, this study underscores the potential of optimized electrode placement to improve simulated TTFields distributions. By leveraging computational modeling and patient-specific anatomical data, the proposed optimization method increased electric field metrics compared to the current clinical standard. These improvements were consistent across various tumor types, sizes, and locations, highlighting the versatility and scalability of the approach. The findings emphasize the potential benefits of personalized TTFields treatment.

Looking forward, integrating adaptive and automated optimization systems could represent important next steps. Continuous monitoring of tumor changes for adjustment of electrode placement during treatment could further enhance the precision and efficacy of TTFields therapies. However, the present study evaluates only simulated electric field distributions, and the relationship between these improvements and therapeutic outcomes remains to be established. Prospective clinical studies will therefore be required to determine whether such optimization strategies translate into measurable clinical benefits.

## Supplementary Material

vdag148_Supplementary_Data

## Data Availability

The datasets generated and analyzed during the current study are not publicly available due to privacy and ethical restrictions but may be shared on request. All shared data will be provided in anonymized form to protect patient confidentiality.

## Code Availability

The approach is available in SimNIBS 4.5 (https://simnibs.github.io/simnibs/build/html/index.html). The optimizations can be carried out on a standard notebook with 16 GB RAM and take about 4-5h per patient to compute.^17^

## References

[1] Kirson ED , Gurvich Z, Schneiderman R, et al Disruption of cancer cell replication by alternating electric fields. Cancer Res. 2004;64:3288-3295. 10.1158/0008-5472.CAN-04-0083 15126372

[2] Mun EJ , Babiker HM, Weinberg U, Kirson ED, Von Hoff DD. Tumor-treating fields: a fourth modality in cancer treatment. Clinic Cancer Res. AACR Inc. 2018;24:266-275. 10.1158/1078-0432.CCR-17-1117 28765323

[3] Stupp R , Taillibert S, Kanner A, et al Effect of tumor-treating fields plus maintenance temozolomide vs maintenance temozolomide alone on survival in patients with glioblastoma. JAMA. 2017;318:2306-2316. 10.1001/jama.2017.18718 29260225 PMC5820703

[4] Leal T , Kotecha R, Ramlau R, et al LUNAR Study Investigators. Tumor treating fields therapy with standard systemic therapy versus standard systemic therapy alone in metastatic non-small-cell lung cancer following progression on or after platinum-based therapy (LUNAR): a randomised, open-label, pivotal phase 3 study. Lancet Oncol. 2023;24:1002-1017. 10.1016/S1470-2045(23)00344-3 37657460

[5] Babiker HM , Picozzi V, Chandana SR, et al PANOVA-3 Study Investigators. Tumor treating fields with gemcitabine and nab-paclitaxel for locally advanced pancreatic adenocarcinoma: randomized, open-label, pivotal phase III PANOVA-3 study. J Clin Oncol. 2025;43:2350-2360. 10.1200/JCO-25-00746 40448572 PMC13472160

[6] Moser JC , Salvador E, Deniz K, et al The mechanisms of action of Tumor Treating Fields. 10.1158/0008-5472.CAN-22-0887/3180540/can-22-0887.pdf PMC9574373 35839284

[7] Wenger C , Miranda PC, Salvador R, et al A review on tumor-treating fields (TTFields): clinical implications inferred from computational modeling. IEEE Rev Biomed Eng. 2018;11:195-207. 10.1109/RBME.2017.2765282 29993870

[8] Mikic N , Gentilal N, Cao F, et al Tumor-treating fields dosimetry in glioblastoma: insights into treatment planning, optimization, and dose–response relationships. Neurooncol Adv. 2024;6:vdae032. 10.1093/noajnl/vdae032 38560348 PMC10981464

[9] Kirson ED , Dbalý V, Tovaryš F, et al Alternating electric fields arrest cell proliferation in animal tumor models and human brain tumors. Proc Natl Acad Sci. 2007;104:10152-10157. 10.1073/pnas.0702916104 17551011 PMC1886002

[10] Ballo MT , Urman N, Lavy-Shahaf G, Grewal J, Bomzon Z, Toms S. Correlation of tumor treating fields dosimetry to survival outcomes in newly diagnosed glioblastoma: a large-scale numerical simulation-based analysis of data from the phase 3 EF-14 randomized trial. Int J Radiat Oncol Biol Phys. 2019;104:1106-1113. 10.1016/j.ijrobp.2019.04.008 31026557

[11] Glas M , Ballo MT, Bomzon Z, et al The impact of tumor treating fields on glioblastoma progression patterns. Int J Radiat Oncol Biol Phys. 2022;112:1269-1278. 10.1016/j.ijrobp.2021.12.152 34963556

[12] Bomzon Z , Hershkovich HS, Urman N, et al Using computational phantoms to improve delivery of Tumor Treating Fields (TTFields) to patients. Paper presented at: 2016 38th Annual International Conference of the IEEE Engineering in Medicine and Biology Society (EMBC). IEEE; 2016:6461-6464. 10.1109/EMBC.2016.7592208 28269726

[13] Wenger C , Salvador R, Basser PJ, Miranda PC. The electric field distribution in the brain during TTFields therapy and its dependence on tissue dielectric properties and anatomy: a computational study. Phys Med Biol. 2015;60:7339-7357. 10.1088/0031-9155/60/18/7339 26350296 PMC4628548

[14] Korshoej AR , Hansen FL, Thielscher A, von Oettingen GB, Sørensen JCH. Impact of tumor position, conductivity distribution and tissue homogeneity on the distribution of tumor treating fields in a human brain: a computer modeling study. PLoS One. 2017;12:e0179214. 10.1371/journal.pone.0179214 28604803 PMC5467909

[15] Korshoej AR , Saturnino GB, Rasmussen LK, von Oettingen G, Sørensen JCH, Thielscher A. Enhancing predicted efficacy of tumor treating fields therapy of glioblastoma using targeted surgical craniectomy: a computer modeling study. PLoS One. 2016;11:e0164051. 10.1371/journal.pone.0164051 27695068 PMC5047456

[16] Opitz A , Windhoff M, Heidemann RM, Turner R, Thielscher A. How the brain tissue shapes the electric field induced by transcranial magnetic stimulation. Neuroimage. 2011;58:849-859. 10.1016/j.neuroimage.2011.06.069 21749927

[17] Weise K , Madsen KH, Worbs T, Knösche TR, Korshøj A, Thielscher A. A leadfield-free optimization framework for transcranially applied electric currents. Comput Biol Med. 2025;195:110648. 10.1016/j.compbiomed.2025.110648 40582165 PMC13284309

[18] Korshoej AR , Thielscher A. Estimating the intensity and anisotropy of tumor treating fields jsing singular value decomposition. Towards a more comprehensive estimation of anti-tumor efficacy. Annu Int Conf IEEE Eng Med Biol Soc (EMBC). IEEE. 2018:4897-4900. 10.1109/EMBC.2018.8513440 30441441

[19] Thielscher A , Antunes A, Saturnino GB. Field modeling for transcranial magnetic stimulation: a useful tool to understand the physiological effects of TMS? Paper presented at: 2015 37th Annual International Conference of the IEEE Engineering in Medicine and Biology Society (EMBC). IEEE; 2015:222-225. 10.1109/EMBC.2015.7318340 26736240

[20] Puonti O , Van Leemput K, Saturnino GB, Siebner HR, Madsen KH, Thielscher A. Accurate and robust whole-head segmentation from magnetic resonance images for individualized head modeling. Neuroimage. 2020;219:117044. 10.1016/j.neuroimage.2020.117044 32534963 PMC8048089

[21] Weller M , van den Bent M, Preusser M, et al EANO guidelines on the diagnosis and treatment of diffuse gliomas of adulthood. Nat Rev Clin Oncol. 2021;18:170-186. 10.1038/s41571-020-00447-z 33293629 PMC7904519

[22] Korshoej AR , Hansen FL, Mikic N, Thielscher A, von Oettingen GB, Sørensen JCH. Exth-04. GUIDING principles for predicting the distribution of tumor treating fields in a human brain: a computer modeling study investigating the impact of tumor position, conductivity distribution and tissue homogeneity. Neuro Oncol. 2017;19:vi73-vi73. 10.1093/neuonc/nox168.300 PMC5467909 28604803

[23] Korshoej AR , Hansen FL, Mikic N, von Oettingen G, Sørensen JCH, Thielscher A. Importance of electrode position for the distribution of tumor treating fields (TTFields) in a human brain. Identification of effective layouts through systematic analysis of array positions for multiple tumor locations. PLoS One. 2018;13:e0201957. 10.1371/journal.pone.0201957 30133493 PMC6104980

[24] Ballo MT , Qualls KW, Michael LM, et al Determinants of tumor treating field usage in patients with primary glioblastoma: a single institutional experience. Neurooncol Adv. 2022;4:vdac150. 10.1093/noajnl/vdac150 36249289 PMC9555297

[25] Ballo MT , Conlon P, Lavy-Shahaf G, Kinzel A, Vymazal J, Rulseh AM. Association of tumor treating fields (TTFields) therapy with survival in newly diagnosed glioblastoma: a systematic review and meta-analysis. J Neurooncol. 2023;164:1-9. 10.1007/s11060-023-04348-w 37493865 PMC10462574

[26] Lok E , San P, Hua V, Phung M, Wong ET. Analysis of physical characteristics of tumor treating fields for human glioblastoma. Cancer Med. 2017;6:1286-1300. 10.1002/cam4.1095 28544575 PMC5463092

[27] Lok E , Clark M, Liang O, Malik T, Koo S, Wong ET. Modulation of tumor-treating fields by cerebral edema from brain tumors. Adv Radiat Oncol. 2023;8:101046. 10.1016/j.adro.2022.101046 36483066 PMC9723310

[28] Panagopoulos DJ , Johansson O, Carlo GL. Evaluation of specific absorption rate as a dosimetric quantity for electromagnetic fields bioeffects. PLoS One. 2013;8:e62663. 10.1371/journal.pone.0062663 23750202 PMC3672148

[29] Wong ET , Lok E. Body fluids modulate propagation of tumor treating fields. Adv Radiat Oncol. 2024;9:101316. 10.1016/j.adro.2023.101316 38260214 PMC10801649

[30] Korshoej AR , Sørensen J, von Oettingen G, Poulsen FR, Thielscher A. Optimization of tumor treating fields using singular value decomposition and minimization of field anisotropy. Phys Med Biol. 2019;64:04NT03. 10.1088/1361-6560/aafe54 30641498

[31] Korshoej AR , Lukacova S, Lassen-Ramshad Y, et al OptimalTTF-1: enhancing tumor treating fields therapy with skull remodeling surgery. A clinical phase I trial in adult recurrent glioblastoma. Neurooncol Adv. 2020;2:vdaa121. 10.1093/noajnl/vdaa121 33215088 PMC7660275

[32] Korshoej AR , Mikic N, Hansen FL, Saturnino GB, Thielscher A, Bomzon Z. Enhancing Tumor Treating Fields Therapy with Skull-Remodeling Surgery. The Role of Finite Element Methods in Surgery Planning. Paper presented at: 2019 41st Annual International Conference of the IEEE Engineering in Medicine and Biology Society (EMBC). IEEE; 2019:6995-6997. 10.1109/EMBC.2019.8856556 31947448

[33] Cao F , Mikic N, Wong ET, Thielscher A, Korshoej AR. Guidelines for burr hole surgery in combination with tumor treating fields for glioblastoma: a computational study on dose optimization and array layout planning. Front Hum Neurosci. 2022;16:909652. 10.3389/fnhum.2022.909652 35782043 PMC9245346

[34] Mikic N , Poulsen FR, Kristoffersen KB, et al Study protocol for OptimalTTF-2: enhancing tumor treating fields with skull remodeling surgery for first recurrence glioblastoma: a phase 2, multi-center, randomized, prospective, interventional trial. BMC Cancer. 2021;21:1010. 10.1186/s12885-021-08709-4 34503460 PMC8427888

[35] Mikic N , Lukacova S, Skjøth-Rasmussen J, et al Dose-enhanced versus standard TTFields for first recurrence of glioblastoma: a randomized phase 2 clinical trial. Neurooncol Adv. 2025;7:vdaf245. 10.1093/noajnl/vdaf245 41473750 PMC12746598

