# Supplementary material for: A new method for optimal placement of tumor treating fields electrodes
Source: Neurooncol Adv. 2026 Jun 3;8(1):vdag148. doi: 10.1093/noajnl/vdag148 (PMC13275299; doi:10.1093/noajnl/vdag148)
Supplement: vdag148_Supplementary_Data [file vdag148_supplementary_data.docx]

**A Method for Optimal Electrode Placement for
Tumor Treating Fields**

***Supplemental Material***

Konstantin Weise^1,2,3*^, Nikola Mikic ^1,4^, Fang Cao^5^, Eric T. Wong^6^,
Thomas R. Knösche^2^, Axel Thielscher^5,7#^, Anders Korshøj^1,4#^

^1^Department of Clinical Medicine, Aarhus University, DK-8200, Aarhus N, Denmark

^2^Methods and Development Group “Brain Networks”, Max Planck Institute for Human Cognitive and Brain Sciences, Stephanstr. 1a, 04103 Leipzig, Germany

^3^Leipzig University of Applied Sciences (HTWK), Wächterstraße 13, 04107 Leipzig, Germany

^4^Department of Neurosurgery, Aarhus University Hospital, DK-8200, Aarhus N, Denmark

^5^Danish Research Centre for Magnetic Resonance, Department of Radiology and Nuclear Medicine, Copenhagen University Hospital Amager and Hvidovre, Nordre Hospitalsvej 13, 2650 Hvidovre, Denmark

^6^Health Cancer Institute, Brown University, Rhode Island, 110 Lockwood St., Providence, RI 02903, USA

^7^Section for Magnetic Resonance, Department of Health Technology, Technical University of Denmark, Ørsteds Plads, building 345C, DK-2800 Kgs. Lyngby, Denmark

* CORRESPONDING AUTHOR

# contributed equally


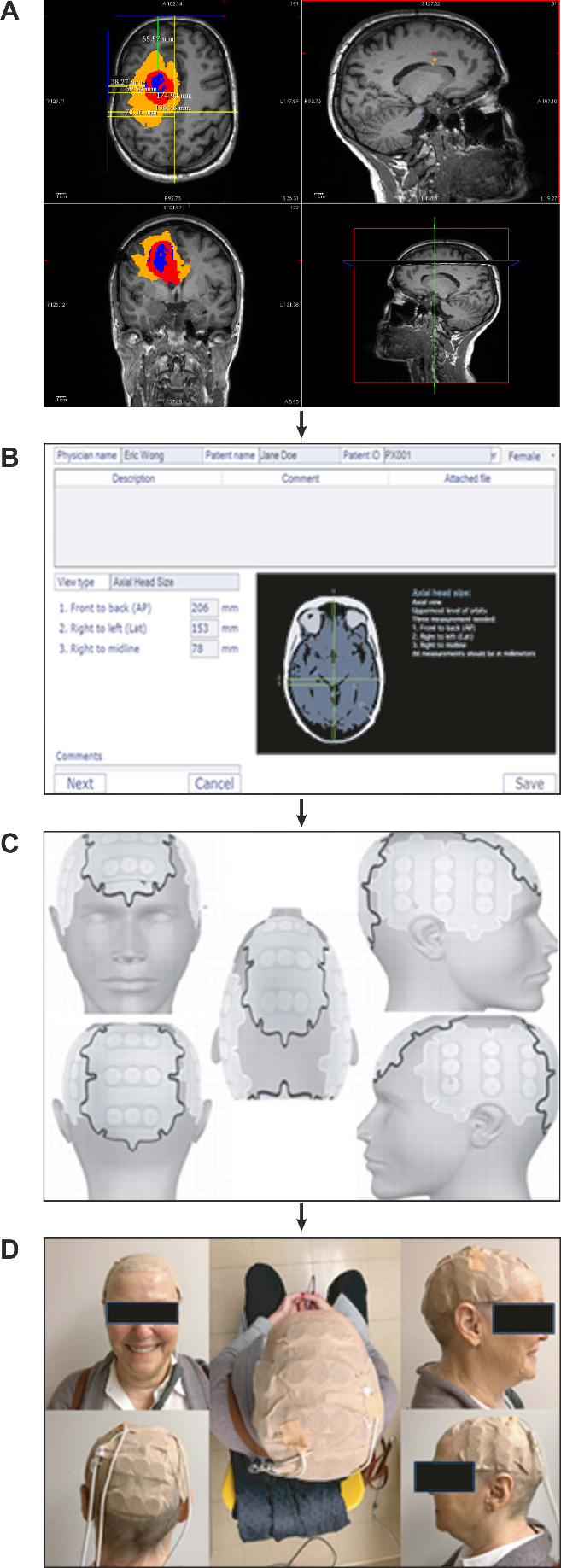


**Fig. S1:** TTFields electrode array planning in a clinical setting using NovoTAL® software (Novocure, Ltd) (A) Head and tumor measurements were taken from MRI DICOM images; (B) The measurements are entered into the NovoTAL® software; (C) The software generates an array layout showing electrode positions on the scalp; (D) The transducer arrays are then applied to the patient according to this map. (modified from Mikic et al. (2024))


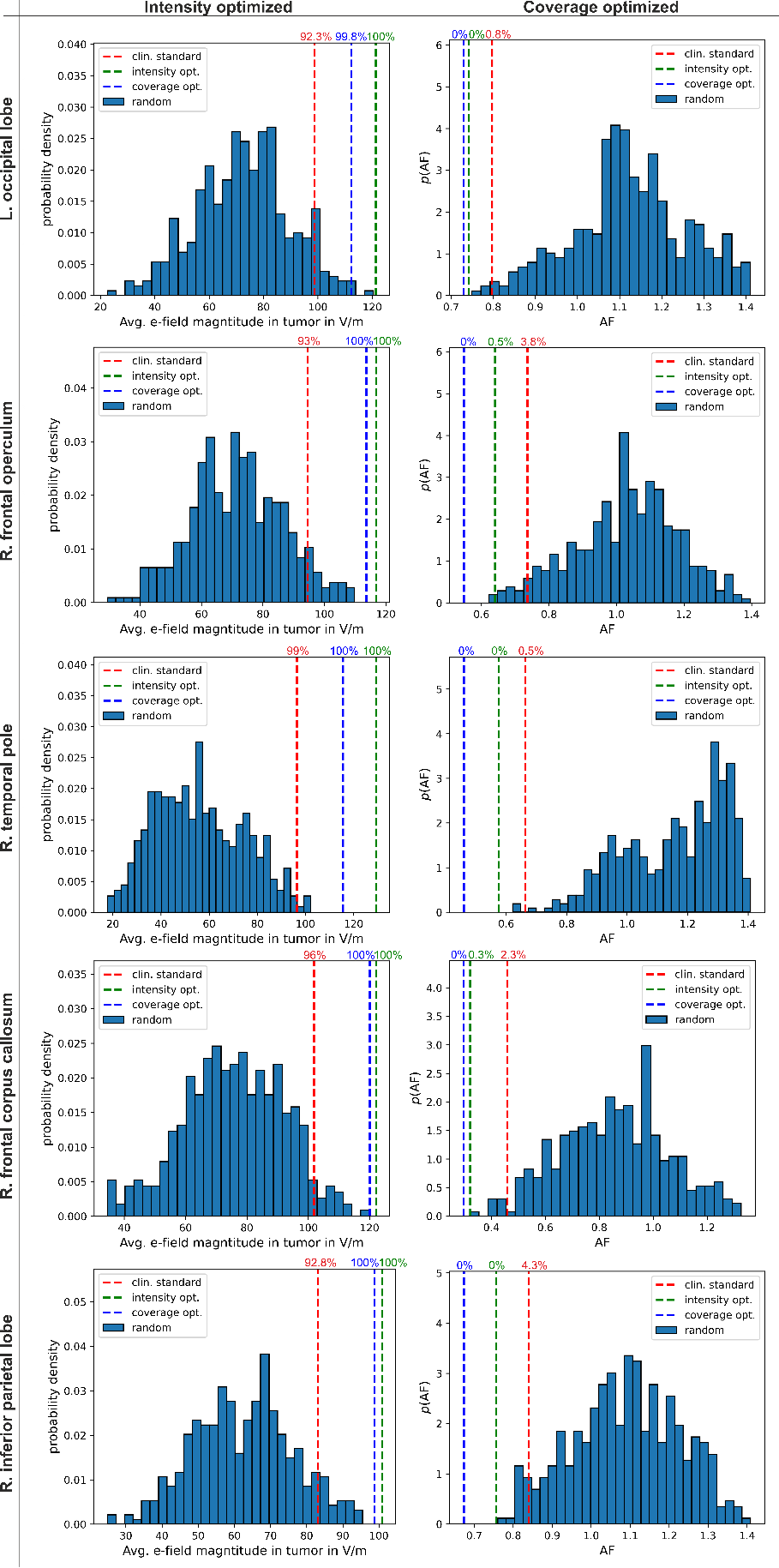


**Fig. S2:** Comparison between 400 random TTFields electrode positions, the current clinical standard and the different optimization techniques (left: e-field intensity optimization, right: e-field spread optimization).

**Table S1:** Tissue types and associated electrical conductivities of the headmodels.

| **Tissue type** | **Electrical conductivity in S/m** | **Reference** |
| --- | --- | --- |
| White matter | $0.126$ | Wagner et al., 2004 |
| Gray matter | $0.275$ | Wagner et al., 2004 |
| Cerebrospinal fluid | $1.654$ | Wagner et al., 2004 |
| Compact bone | $0.008$ | Opitz et al., 2015 |
| Spongy bone | $0.025$ | Opitz et al., 2015 |
| Eyes | $0.500$ | Opitz et al., 2015 |
| Blood | $0.600$ | Gabriel et al., 2009 |
| Muscle | $0.160$ | Gabriel et al., 2009 |
| Skin | $0.465$ | Wagner et al., 2004 |
| Residual tumor | $0.24$ | Korshoej et al. 2017  Korshoej et al. 2018 |
| Necrosis | $1.0$ | Korshoej et al. 2017  Korshoej et al. 2018 |
| Edema | $0.71$ | Korshoej et al. 2017  Korshoej et al. 2018 |

**References**

Gabriel, C., Peyman, A., & Grant, E. H. (2009). Electrical conductivity of tissue at frequencies below 1 MHz. Physics in medicine & biology, 54(16), 4863.

Korshoej, A. R., Hansen, F. L., Thielscher, A., von Oettingen, G. B., & Sørensen, J. C. H. (2017). Impact of tumor position, conductivity distribution and tissue homogeneity on the distribution of tumor treating fields in a human brain: A computer modeling study. PloS one, 12(6), e0179214.

Korshoej, A. R., Hansen, F. L., Mikic, N., von Oettingen, G., Sørensen, J. C. H., & Thielscher, A. (2018). Importance of electrode position for the distribution of tumor treating fields (TTFields) in a human brain. Identification of effective layouts through systematic analysis of array positions for multiple tumor locations. PLoS One, 13(8), e0201957.

Mikic, N., Gentilal, N., Cao, F., Lok, E., Wong, E. T., Ballo, M., ... & Korshoej, A. R. (2024). Tumor-treating fields dosimetry in glioblastoma: Insights into treatment planning, optimization, and dose–response relationships. Neuro-Oncology Advances, 6(1), vdae032.

Opitz, A., Paulus, W., Will, S., Antunes, A., & Thielscher, A. (2015). Determinants of the electric field during transcranial direct current stimulation. Neuroimage, 109, 140-150.

Wagner, T. A., Zahn, M., Grodzinsky, A. J., & Pascual-Leone, A. (2004). Three-dimensional head model simulation of transcranial magnetic stimulation. IEEE Transactions on Biomedical Engineering, 51(9), 1586-1598.
